# Tox21 to Date: Steps toward Modernizing Human Hazard Characterization

**DOI:** 10.1289/ehp.121-a228

**Published:** 2013-07-01

**Authors:** Kellyn S. Betts

**Affiliations:** Kellyn S. Betts writes about environmental contaminants, hazards, and technology for solving environmental problems for publications including *EHP* and *Environmental Science & Technology*.

A new review in *EHP* describes the first phase of the federal government’s Tox21 collaboration, which is attempting to establish alternatives to the time-consuming and expensive animal testing used to evaluate chemical toxicity.^1^ The review details important strides investigators have made in demonstrating the usefulness of high-throughput data for identifying potential hazards and prioritizing chemicals for more extensive testing. In the long run, the program may help “protect people from exposure to a much greater number of harmful chemicals than is possible based on current testing methods,” says lead author Raymond Tice, chief of the Biomolecular Screening Branch of the NIEHS’s National Toxicology Program (NTP) Division.

Officially begun five years ago, Tox21 is an outgrowth of U.S. federal interagency collaborations initiated in 2005 to explore the utility of a high-throughput screening program. It includes scientists at NTP and other NIH entities as well as the Environmental Protection Agency’s (EPA) National Center for Computational Toxicology. In 2010 the Food and Drug Administration (FDA) joined Tox21, but the new report focuses mainly on the efforts of the original Tox21 partners.

“At the moment, I think Tox21 is clearly the most important effort to revamp toxicology,” says Thomas Hartung, who directs the Center for Alternatives to Animal Testing (CAAT), part of the Johns Hopkins Bloomberg School of Public Health. He considers the number of substances included, the quality assurance applied, and the public availability of data from Tox21 unique.

Tox21 mainly uses cell-based and biochemical *in vitro* tests, although some testing is conducted with lower animals such as *Danio rerio* (zebrafish) and *Caenorhabditis elegans* (a nematode species). In the first phase of testing at the NIH Chemical Genomics Center, scientists screened up to 2,800 compounds with about 70 high-throughput assays.^1^ Tice points out that the number of compounds screened is relatively small from the perspective of the tens of thousands that need screening. But he says this number was adequate to demonstrate the utility of the approach and to identify assay-specific limitations.

Phase I focused on evaluating how assays performed and the extent to which they could be optimized. The percentage of compounds classified as active by the assays ranged from 0.07% for an epigenetics cell-based assay to 41% for an assay that tested the ability of compounds to interact with the cytochrome P450 enzyme CYP1A2, which is involved in the metabolism of foreign substances.^1^

**Figure:**
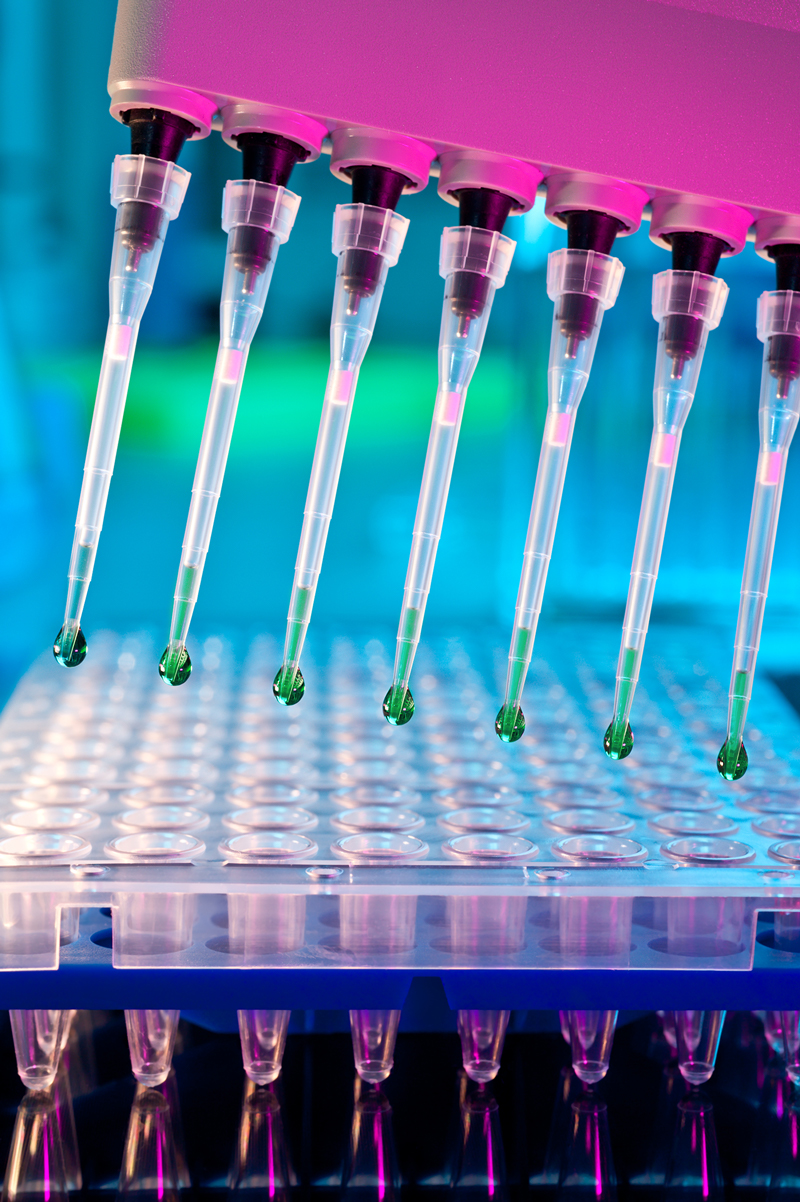
Phase I of Tox21 has provided insight into possible ways of optimizing highthroughput screening. © anyaivanova/Shutterstock

Lorenz Rhomberg, a principal for the environmental consulting firm Gradient, says he believes the review is most important for describing the limitations of Tox21’s first phase, especially the fact that although thousands of chemicals can be screened in a single assay, it is difficult to interpret the patterns of changes in terms of particular pathways of toxicity generation. In addition, Rhomberg says, the chosen assays may not cover all the toxicological outcomes of potential concern that are currently evaluated in whole-animal studies.

Problems identified during Phase I are being addressed in Phase II, which is now in full swing. According to the authors, the primary limitation of Tox21’s quantitative high-throughput testing in Phase I was that the biological output of each of the assays currently used was generally limited to one or two “signals” (measurable responses). Investigators have begun developing cell-based assays with hundreds to thousands of signals. And because Tice and his colleagues discovered that some of the compounds they purchased for testing had inaccurate information on their certificates of analysis or were unstable during storage and use, Phase II includes analysis of test chemicals for identity, purity, and stability.

Another issue that came to light was what Tice calls “compound carryover”—some compounds can be transferred from one set of plates to another by the pin tools used in the quantitative high-throughput screening system. To compensate for this problem, the investigators are screening each substance at least three times in Phase II, changing the compounds’ well locations in each run.

Another important limitation that Tice and his colleagues are trying to remedy for Tox21’s next phase is the inability of most cell lines to mimic *in vivo* metabolism of chemicals, which can produce intermediates that are more biologically active than the parent compound. Because the liver is the primary site where such reactive intermediates are metabolized, the Tox21 researchers are investigating whether cells with hepatocyte functionality can be used to assess a chemical’s ability to interact with multiple cellular pathways.

The investigators stress that they still face many obstacles before they can definitively show that Tox21 will indeed transform toxicity testing. But they nonetheless believe the data and information they are obtaining is “foretelling the future of toxicology.”
